# Better, worse, or different than expected: on the role of value and identity prediction errors in fear memory reactivation

**DOI:** 10.1038/s41598-022-09720-w

**Published:** 2022-04-07

**Authors:** A. M. V. Gerlicher, S. A. Verweij, M. Kindt

**Affiliations:** Department of Clinical Psychology, University of Amsterdam, Nieuwe Achtergracht 129B, 1018WS Amsterdam, The Netherlands

**Keywords:** Human behaviour, Learning and memory, Fear conditioning, Anxiety

## Abstract

Although reconsolidation-based interventions constitute a promising new avenue to treating fear and anxieties disorders, the success of the intervention is not guaranteed. The initiation of memory reconsolidation is dependent on whether a mismatch between the experienced and predicted outcome-a prediction error (PE)-occurs during fear memory reactivation. It remains, however, elusive whether any type of PE renders fear memories susceptible to reconsolidation disruption. Here, we investigated whether a value PE, elicited by an outcome that is better or worse than expected, is necessary to make fear memories susceptible to reconsolidation disruption or whether a model-based identity PE, i.e., a PE elicited by an outcome equally aversive but *different* than expected, would be sufficient. Blocking beta-adrenergic receptors with propranolol HCl after reactivation did, however, not reduce the expression of fear after either type of PE. Instead, we observed intact fear memory expression 24 h after reactivation in the value-, identity- and a no-PE control group. The present results do not corroborate our earlier findings of reconsolidation disruption and point towards challenges that the field is currently facing in observing evidence for memory reconsolidation at all. We provide potential explanations for the unexpected failure of replicating reconsolidation disruption and discuss future directions.

## Introduction

Reconsolidation-based interventions constitute a promising new avenue for the treatment of fear, anxiety and trauma-related disorders^1,2^. They are rooted in the neuroscientific (re)discovery that reactivating a stable fear memory can convert it back into a labile, malleable state that is thought to allow for the integration of new information^3–5^. Once the fear memory is destabilized, a time-dependent protein-synthesis dependent process, termed memory reconsolidation, is necessary to re-stabilize it and allow it to further persist^5^. This creates a unique window of opportunity during which the administration of a protein synthesis inhibitor or other pharmacological agents can disrupt the memory reconsolidation process and induce post-reactivation amnesia for the destabilized fear memory^4^. First demonstrated in rodents, reconsolidation-based interventions have been shown to be effective in reducing the affective component of fear memories in healthy humans^6–11^ and subsequently in phobic participants as well^12^. In contrast to exposure-based treatments, reconsolidation-based interventions do not rely on new inhibitory learning but rather weaken the original fear memory itself. For this reason, fear does not return after a reconsolidation-based intervention upon classical laboratory challenges of relapse, such as reinstatement, renewal or spontaneous recovery^13^. Despite the great potential this new approach bears for the treatment of fear and anxiety disorders, the exact conditions that render human fear memories labile and susceptible to reconsolidation disruption are not yet fully understood^2^.

Past research has shown that a prediction error (PE)-or a mismatch between what actually occurs and what is expected-is required to destabilize a previously consolidated memory trace^14^. As an example, in a fear-conditioning paradigm a conditioned stimulus (CS) that is followed by an outcome (an unconditioned stimulus, US) that is *better* or *worse* than expected elicits a PE and renders this fear memory susceptible to reconsolidation disruption^9^. Different reinforcement learning theories postulate that such value-PEs (i.e., mismatches in outcome value) drive new learning^15,16^. On an algorithmic level^17^, this form of learning could be implemented by so-called ‘model-free’ learning^18,19^. Model-free learning learns about the expected value of stimuli (i.e., stimulus is associated with reward, stimulus is associated with threat) from the *actual experience* of value-PEs^18,20^. As it only learns from value-mismatches and does not rely on the creation of any ‘model’ or representation of the environment, model-free learning is computationally simple. However, in absence of any actual experience or absence of any value differences in outcomes, no model-free learning would take place. In order to account for these limitations, it is suggested that model-free learning is complemented by so-called ‘model-based’ learning^18,19^. Model-based learning allows for the creation of an internal model or representation of the environment. It can learn from value-PEs, but also from mismatches in sensory properties of the outcome or changes in the outcome identity^21^ and update predictions instantaneously upon new information or changes in the environment, without the need for actual experience. Initially described and fruitfully investigated in the context of instrumental learning^22^, there is evidence that both model-free and model-based learning are implemented in the brain^23^ and play a role in Pavlovian conditioning^19^. Hence, the question arises whether model-based learning also contributes to the destabilization of fear memories in the context of reconsolidation-based interventions.

We can look at the conditions that destabilize or do not destabilize fear memories through the lens of model-free and model-based learning processes. As stated above, when outcomes are clearly better or worse than expected, a ‘value PE’ in both a model-free and model-based system is elicited and fear memories are destabilized and become susceptible to reconsolidation-intervention^9^. Similarly, it was shown that changes in the temporal relationship between CS and US allow for reconsolidation disruption in animals^24^. Such temporal PEs are also registered in both model-free (e.g., Rescorla-Wagner or temporal-difference learning^15,16^) and model-based learning^16^. Interestingly, however, an unreinforced presentation of a CS in the absence of the US electrode was not able to destabilize fear memories^8^. In a model-free learning system, the absence of the US electrode would not (fully) reduce the US prediction and the US omission would still elicit a model-free PE. In contrast, model-based learning can integrate information about the absence of the electrode and can instantaneously update the probability of experiencing the US to zero, thus, no model-based PE would be elicited when the electrode is detached. The observation that reconsolidation disruption was not successful when the US-electrode was detached^8^ therefore suggests that a model-free PE *alone* is insufficient to successfully destabilize fear memories. Based on these results (for visualization see Supplementary Table 1), one could therefore postulate as a working hypothesis that the *co-occurrence* of model-free and model-based PEs is necessary to make fear memories susceptible to reconsolidation disruption. However, up to date it has not been tested yet whether a model-based PE on its own could destabilize fear memories.

**Figure 1:**
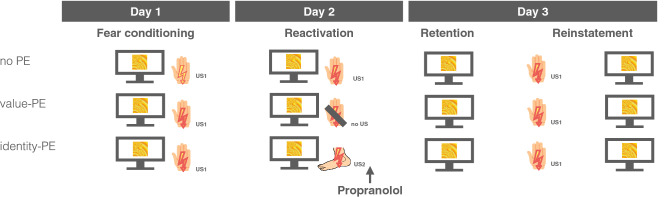
Experimental design. All groups underwent fear conditioning on day 1 during which a visual stimulus (CS+) was reinforced with an electric stimulus to either wrist or ankle (US1), whereas another CS (CS−) was never paired with the US (not shown). During memory reactivation on day 2, the ‘no PE’ or control group was presented with the same CS+ US1 pairing again. The US1 was omitted in the value-PE group eliciting both a model-free and model-based prediction error. In the identity-PE group the CS+ was paired with an electric stimulus to the respective other location (US2) eliciting a model-based identity, but no model-free PE. Subsequently, all groups received 40 mg propranolol HCl. We tested the effect of this reconsolidation intervention with different PE manipulations on fear potentiated startle responses during retention and after reinstatement on day 3.

Understanding the exact conditions that render fear memories labile is important to inform the translation of reconsolidation-based interventions into clinical practice^2^. For this reason, the present study aimed to address the question whether a purely model-based PE destabilizes a fear memory by manipulating the identity of the US during memory reactivation. Specifically, we used a three-day human fear conditioning paradigm with two aversive electric stimuli, one to the wrist and one to the ankle of the participant (see Fig. 1). Before the start of the experiment, we calibrated each electric stimulus to a level rated as maximally uncomfortable and equally uncomfortable as the stimulus to the respective other body location. One of the electric stimuli was subsequently used as unconditioned stimulus (US1) and employed as reinforcer of a CS (CS+) during conditioning on day 1. Another CS remained unreinforced and served as control stimulus (CS−). Before memory reactivation on day 2, participants were assigned to one of three groups. During reactivation, one group was presented with a pairing of the CS+ and the US1, as on day 1. We reasoned that the CS+ US1 pairing should be fully predicted after conditioning with 100% reinforcement on day 1 and, thus, not elicit a PE (no PE, control group). In a second group, the CS+ was not followed by the US1 (value-PE group). After conditioning with 100% reinforcement the sudden omission of the US1 should elicit a model-free and model-based value PE and render the fear memory labile and susceptible to pharmacological reconsolidation disruption. Lastly, in a third group, the CS+ was unexpectedly reinforced by the US2 (identity-PE group). Due to the matching of the USs in terms of aversiveness, the delivery of the US2 should not elicit a model-free PE, but the mismatch in outcome identity would be registered as ‘identity’ PE in the model-based learning system only. Subsequently, all groups received 40 mg propranolol HCl and we tested the effect of the combination of the three PE manipulations and propranolol on differential startle responses during a retention and reinstatement test on day 3.

We predicted that the recruitment of both model-free and model-based learning is necessary to trigger memory reconsolidation. Thus, the unexpected omission of the US1 in the value-PE group should destabilize the fear memory and make it susceptible to a disruption of fear memory reconsolidation by propranolol. Evidence for these non-observable neurobiological processes would be inferred from a lower fear response compared to the no-PE control group at test 24 h later. Propranolol administration after a model-based identity PE is, however, likely to be insufficient for fear memory reactivation. Thus, the identity-PE group was predicted to show higher fear responding than the value-PE group. Alternatively, if a model-based PE alone would be sufficient to destabilize the memory trace, propranolol administration should interfere with fear memory reconsolidation in the identity-PE group as well. In that case, we should also observe reduced differential fear responding during both retention and reinstatement rest in the identity-PE compared to the no-PE control group.

## Methods

### Participants

A total of N = 60 individuals (18/42 male/female, mean ± SD 20.64 ± 2.46 years, range 18–29 years) participated in the experiment. Participants gave written informed consent and were screened to be free from conditions contraindicating the intake of propranolol HCl^25^. Participants were randomly assigned to the control group (N = 20, 14 female), the value-PE group (N = 20, 14 female), or the identity-PE group (N = 20, 14 female) with the restriction that groups were matched on questionnaire scores of the State-Trait Anxiety Inventory-Trait version (STAI-T^26^) and the Anxiety Sensitivity Index (ASI-3^27^; see Table 1). Participants received either course credits or a financial reimbursement (€50) for participation. The study was approved by the Ethical Review Board of the University of Amsterdam and conducted in accordance with the Declaration of Helsinki.

**Table 1:** Demographic information, mean (standard deviation, SD) of STAI-T and ASI questionnaire scores, US intensities and US intensity ratings (pre- and post-conditioning) for participants in the control, the value-PE and identity-PE group.

	no-PE	value-PE	identity-PE	*F*	*P*-value
Age	21.1 (2.9)	20.9 (2.6)	19.9 (1.7)	1.35	.27
STAI-T	37.2 (7.0)	39.7 (8.9)	37.7 (8.4)	.53	.59
ASI-3	14.8 (7.9)	13.4 (8.6)	15.2 (10.3)	.23	.80
US arm (mA)	42.4 (19.9)	43.4 (21.1)	38.5 (21.4)	.31	.73
US leg (mA)	60.2 (24.4)	56.7 (26.1)	50.4 (25.4)	.78	.46
US1 rating pre	8.9 (.34)	9.0 (.00)	8.8 (.67)	.82	.44
US1 rating post	8.6 (.61)	8.5 (.68)	8.6 (.74)	.06	.94
US2 rating pre	8.9 (.34)	9.0 (.22)	8.8 (.67)	.43	.66
US2 rating post	8.7 (.81)	8.7 (.75)	8.7 (.73)	.01	.99

There were no significant group differences as indicated by ANOVA with between-subject factor ‘group’ (F- and *P*-values).

### Sample size estimation

The required sample size was estimated using ‘G*Power’^28^. We estimated the to-be-expected effect size based on a previous study^9^ that investigated the effect of different PE manipulations on fear memory reactivation, specifically, the result that the three groups differed in startle responding on the first retention trial of extinction on day 3 (stimulus × group: *F*_2,42_ = 6.49, *P* < 0.003, *η*^2^_*p*_ = 0.24). In that study, we observed a large effect of *η*^2^_*p*_ = 0.24 or *f* = 0.56. Together with a power 1-β = 0.80 and an alpha-level of *P* = 0.05 this effect size yielded a required sample size of N = 29. As (partial) replications often show substantially smaller effect sizes^29^ and in order to account for the noisiness of physiological measures, we increased the sample size to N = 60 (i.e. N = 20 per group).

### Stimuli

In contrast to previous studies^6–11,25,30,31^, we employed two neutral instead of fear relevant images as CSs in the present experiment. This decision was based on feasibility considerations for data collection during the COVID-19 pandemic. Participants with high fear of spiders need to be excluded when fear relevant spider images are used which reduces the number of available participants considerably. In the present study, two abstract fractals (yellow, blue) served as CS+ and CS− instead. Assignment of the yellow and blue fractal to CS+ /CS− was randomized between participants and matched between groups.

### Unconditioned Stimuli

An electric stimulus to either the left wrist or the left ankle served as US1 and US2, respectively. Assignment of stimulus location (wrist, ankle) to US1 and US2 was randomized between participants and counter-balanced between groups. Electric stimuli were delivered via two Ag/AgACl electrodes of 20 by 25 mm with fixed inter-electrodes mid-distances of 45 mm and prepared with conductive-gel (Signa Gel, Parker Laboratories Inc.). The delivery of the electric stimuli was controlled by a Digitimer DS7A (Digitimer, Weybridge). Each electric stimulus consisted of a single square-wave pulse with a duration of 2 ms. Before the start of the experiment the intensities of the electric stimuli were calibrated individually to a level judged as ‘maximally uncomfortable, but not yet painful’ by the participant on a rating scale (0 = ‘I do not feel anything’, 5 = ‘medium uncomfortable’, 10 = ‘already painful’). After the calibration of the wrist and ankle stimulus, we presented participants with both electric stimuli again and asked them to judge whether they were equally strong or whether one was perceived as stronger than the other. If participants indicated that one was stronger than the other, we recalibrated the weaker stimulus until it matched the stronger in perceived intensity. The intensity ratings were repeated after conditioning on day 1. Group means of electric stimulus intensity and pre- and post-conditioning intensity ratings of the US1 and US2 are provided in Table 1.

### Pre- and post-session US expectancy ratings

We decided to employ pre- and post-session (i.e., ‘offline’) US expectancy ratings in the present study as conducting two different ratings (US1, US2) during each trial risked diverting attention away from the CS, which could in turn have affected learning^32,33^. Thus, participants were asked to indicate their expectancy before the start and after the end of each experimental session. They rated their expectancy to receive an electric stimulus to either wrist or ankle for each CS on a scale ranging from 0–100 (0 = no expectancy, 100 = high expectancy) with a mouse-click.

### Fear potentiated startle response (FPS)

The startle reflex was elicited by a loud noise (40 ms; 104 dB) presented binaurally via headphones (Model MD-4600; Compact Disk Digital Audio, Monacor) during CS+ and CS− and noise alone (NA) trials. Electromyographic (EMG) activity was recorded using 7 mm Ag/AgCl electrodes filled with conductive gel and positioned approximately 1 cm below the pupil and 1 cm below the lateral canthus, the outer corner of the eye^34^. A ground electrode was placed on an electrically neutral site on the forehead. The EMG signal was amplified and digitized at 1000 Hz. Before preprocessing, the EMG data was visually screened for recording artifacts. Day 1 and day 2 FPS data of N = 2 participants needed to be excluded due to problems with the recording software (i.e., no signal recorded). Their FPS data of day 3 were, however, complete and included in the analysis. For reasons of transparency, we also present the results of day 3 after exclusion of these participants in the Supplementary Material (note, excluding them did not change the results). The signal was analyzed offline using Psycho-Physiological Modelling (PsPM 5.0.0^35^ in Matlab 2020a, Mathworks ®, Natrick, Massachusetts, USA). In PsPM, the signal was band-pass filtered (cut-off: 50 Hz and 470 Hz, 4th order Butterworth filter). Furthermore, a notch filter was applied to remove 50 Hz noise. The resulting signal was smoothed using a low-pass filter (cut-off: 53.05 Hz, 4th order Butterworth filter), rectified and down-sampled to 500 Hz^36^. We employed a single-trial general linear model (GLM) to estimate trial-by-trial startle responses. The model comprised one individual regressor for each startle-probe onset convolved with a canonical startle response function with a flexible response onset latency of 0–100 ms. For statistical analysis, single-trial parameter estimates were Z-transformed within each participant across stimuli (CS+ , CS−, NA; excluding habituation trials) and sessions.

### Drug treatment

Participants received an oral dose of 40 mg of propranolol directly after memory reactivation in a single-blind fashion (i.e., the participants were blind to whether they received a placebo or propranolol pill even though all participants received the active drug). Peak plasma levels of propranolol are reached 1–2 h after intake^37^ allowing a post-reactivation administration of propranolol to successfully interfere with fear memory reconsolidation^7,9,11,31,38^.

### Experimental procedure

#### Day 1-conditioning

Upon arrival participants filled out informed consent and a medical screening form. Furthermore, heart rate and blood pressure were assessed to confirm eligibility for propranolol administration (i.e., resting heart rate > 50 bpm, blood pressure > 100/60 mmHg). Participants filled in the ASI and STAI-T/S questionnaires. Subsequently, the experimenter attached the EMG and electric stimulus electrodes and calibrated the intensity of the two electric stimuli. Before the start of the experiment, participants were instructed that they would be presented with either a yellow or a blue image on the computer screen, one of which would sometimes be followed by a shock, while the other, would never be followed by a shock, on none of the three days. Before the start of conditioning, participants rated their expectancy to receive the arm or leg shock for CS+ and CS−, respectively. To ensure startle responses habituation, participants were presented with 10 noise alone (NA) trials. During conditioning 5 CS+, 5 CS− and 5 NA trials were presented. Trial order was randomized in such a way that not more than two trials of the same type succeeded each other. The CS duration was 6500 ms. The startle probe was presented 6000 ms after CS onset and US delivery occurred 6450 ms after CS onset. All 5 out of 5 CS+ trials were reinforced with a US (i.e., 100% reinforcement). Inter-trial intervals (ITI) lasted 17.5 s on average (range: 15–20 s). After conditioning, electrodes were detached and participants were instructed to not smoke, eat or drink anything else than water 2 h before the second appointment the following day. Additionally, they were instructed to not consume alcohol or drugs that night and warned that we could conduct drug urine tests on a random sample of participants.

#### Day 2-reactivation

Approximately 24 h (± 2 h) after conditioning, participants returned to the laboratory. Blood pressure and heart rate were assessed to ensure eligibility for propranolol intake (i.e., resting heart rate > 50 bpm, blood pressure > 100/60 mmHg). After attachment of the electrodes, the participants were instructed that the experiment would simply continue, and they should remember what they had learned the day before. The experiment started with US expectancy ratings and 10 NA habituation trials. During memory reactivation, participants were presented with one CS+ trial and one NA trial. The CS+ was either as on day 1 reinforced with the US1 (no PE, control group), not reinforced (value-PE group), or reinforced with the US2 (identity-PE group). All participants received 40 mg propranolol HCl directly after reactivation. Subsequently, participants stayed in the laboratory under supervision for 90 min and blood pressure and heart rate were assessed 90 min after pill intake again (see Supplementary Table 2). Before leaving the laboratory, participants were instructed to not consume alcohol or drugs that night and warned that we could conduct drug urine tests on a random sample of participants.

#### Day 3-retention and reinstatement test

Approximately 24 h (± 2 h) after reactivation, participants returned to the laboratory for a memory retention and reinstatement test. After attachment of the electrodes, the participants were instructed that the experiment would simply continue, and they should remember what they had learned the day before. The experiment started again with US expectancy ratings and 10 NA habituation trials. For the retention test, participants were presented with 16 CS+ , CS− and NA trials in extinction, i.e., CS+ was not reinforced with US1 or US2 in any of the three groups. To test a potential reinstatement of the original fear memory trace, we subsequently delivered three unannounced US and tested reinstatement on 1 CS+ , CS− and NA trial. After the end of the test session, participants completed US expectancy ratings and answered questions regarding the CS–US contingencies and the subjective intensity of US1 and US2.

### Statistical analysis

All statistical analyses were performed in RStudio^39^ (v1.1456). Analyses of fear-potentiated startle responses (FPS) followed a previous study that employed three different PE manipulations^9^. To test whether conditioning was successful on day 1, we compared FPS of the first to the last trial of conditioning using a repeated-measures ANOVA (rmANOVA) with stimulus (CS+, CS−) and trial (trial 1, trial 5) as within-, and group (no-PE, value-PE, identity-PE) as between-subject factors. To assess whether conditioned responding was intact during memory reactivation on day 2, we compared FPS to the CS+ and NA trial using a rmANOVA with stimulus (CS+, NA) as within- and group (no-PE, value-PE, identity-PE) as between-subject factor. To test the hypotheses, we assessed whether the three groups differed on differential FPS (CS+  > CS−) during the first retention trial of extinction on day 3 using a rmANOVA with stimulus (CS+, CS−) as within- and group (no-PE, value-PE, identity-PE) as between-subject factor. We assessed whether extinction differed between groups using a rmANOVA with stimulus (CS+, CS−) and trial (first, last) as within- and group (no-PE, value-PE, identity-PE) as between-subject factor on the FPS data on day 3. Lastly, we tested whether there were group differences on the trial after reinstatement on day 3 using a rmANOVA with stimulus (CS+, CS−) as within- and group (no-PE, value-PE, identity-PE) as between-subject factor. All ANOVAs were computed using the ez-package^40^ (v4.4.0) and Type-III sum of squares. Results were considered statistically significant when *P* < 0.05 (two-sided tests).

## Results

### Manipulation check–US ratings pre- and post-conditioning

In order to ensure that the delivery of the US2 at memory reactivation would primarily elicit an identity but not a value PE in the identity-PE group, we had calibrated the two USs before the start of the experiment until they were perceived as equally uncomfortable (mean US1: 8.92, mean US2: 8.90; US: *F*_*1,57*_ = 0.1.00, *P* = 0.32, group: *F*_*2,57*_ = 0.60, *P* = 0.55, US × group: *F*_*2,57*_ = 0.1.00, *P* = 0.37; for group means see Table 1). Directly after conditioning on day 1, we asked participants to rate the aversiveness of both USs again. The aversiveness ratings decreased slightly but significantly from before to after conditioning for both US1 and the US2 (time: *F*_*1,57*_ = 23.65, *P* < 0.001, *η*^*2*^_*p*_ = 0.29, time × US: *F*_*1,57*_ = 1.79, *P* = 0.19) in all groups (all *F*’s < 0.92, all *P’*s > 0.40). Thus, even though only the US1 was presented during conditioning and the aversiveness decreased over the course of conditioning for the two USs, both USs were still rated as equally uncomfortable after conditioning (mean US1: 8.57, mean US2: 8.66; US: *F*_*1,57*_ = 1.21, *P* = 0.28) in all groups (all *F*’s < 0.04, all *P’*s > 0.96).

### Manipulation check–Effect of propranolol on blood pressure and heart rate

From before to after the intake of propranolol HCl on day 2, we observed the to-be-expected significant decrease of both systolic and diastolic blood pressure (systolic: *F*_*1,57*_ = 29.31, *P* < 0.001, *η*^*2*^_*p*_ = 0.34; diastolic: *F*_*1,57*_ = 8.67, *P* = 0.005, *η*^*2*^_*p*_ = 0.13) that did not differ significantly between groups (stimulus × group: all *F*’s < 0.74, all *P’*s > 0.48; group: all *F*’s < 1.20, all *P’*s > 0.30; Supplementary Table 2). The average systolic (mean: − 13.67 mmHg) and diastolic (mean: − 4.10 mmHg) blood pressure was comparable to decreases observed in propranolol-treated participants in previous studies (see Supplementary Table 3). Similarly, propranolol HCl intake decreased heart rate significantly (mean: − 20.33 bpm*; F*_*1,57*_ = 159.01, *P* < 0.001, *η*^*2*^_*p*_ = 0.74) in all groups (stimulus × group: *F*_*2,57*_ = 0.10, *P* = 0.91; group: *F*_*2,57*_ = 0.04, *P* = 0.96).

### US expectancy ratings

#### Day 1-aversive conditioning

**Figure 2:**
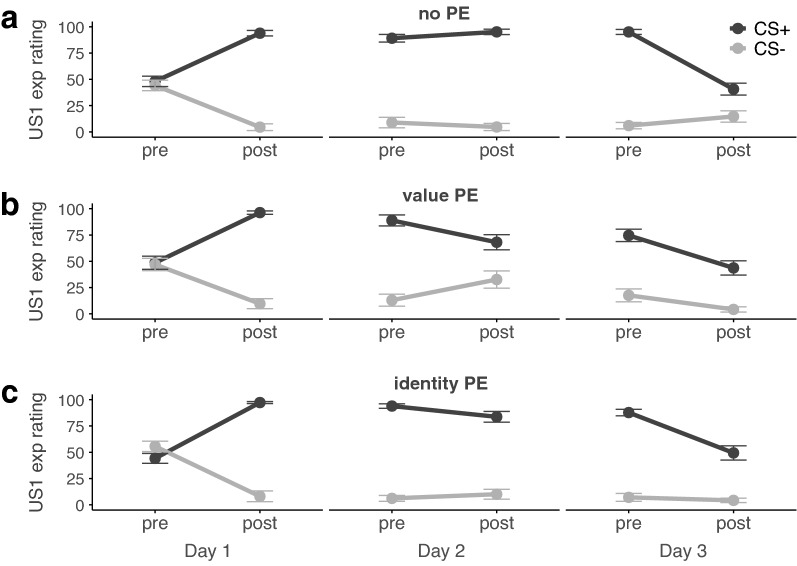
Pre- and post-session expectancy to receive the US1 for CS+ and CS− in (**a**) the no-PE control group, (**b**) the value-PE group for which the US1 was omitted during memory reactivation on day 2, and (**c**) the identity-PE group for which the CS+ was paired with the US2 during memory reactivation on day 2. Error bars depict standard error of the mean.

**Figure 3:**
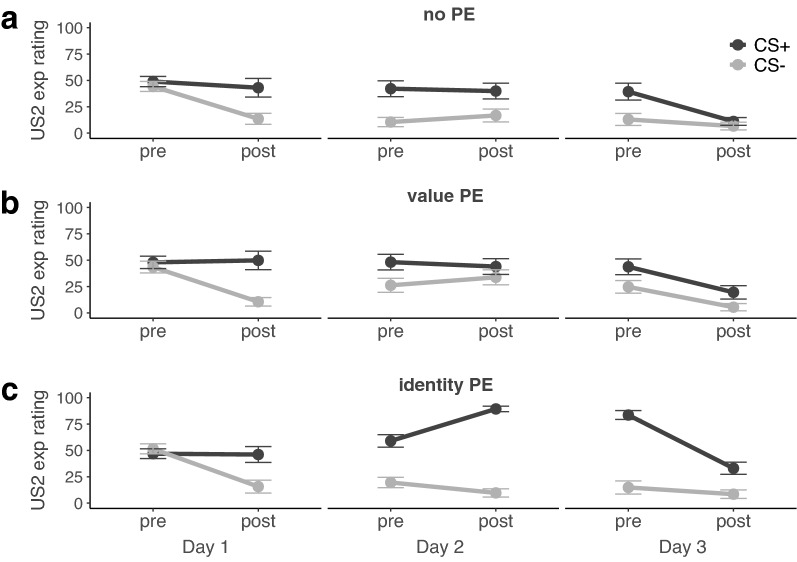
Pre- and post-session expectancy to receive the US2 for CS+ and CS− in (**a**) the no-PE control group, (**b**) the value-PE group for which the US1 was omitted during memory reactivation on day 2, and (**c**) the identity-PE group for which the CS+ was paired with the US2 during memory reactivation on day 2. Error bars depict standard error of the mean.

Before and after each experimental session, participants rated their expectancy to receive an electric stimulus to either wrist or ankle for the CS+ and CS−, respectively. Expectancy ratings confirmed that participants successfully acquired the contingency between CS+ and US1 as indicated by a significant stimulus × US × time interaction (*F*_*1,56*_ = 69.91, *P* < 0.001, *η*^*2*^_*p*_ = 0.56) that did not differ between groups (stimulus × US × time × group: *F*_*1,56*_ = 0.42, *P* = 0.66; see Fig. 2; for complete results see Supplementary Table 4). Follow-up tests showed that expectancies to receive the US1 significantly increased from before to after conditioning for the CS+ in all groups (time: *F*_*1,56*_ = 240.94, *P* < 0.001, *η*^*2*^_*p*_ = 0.81; group: *F*_*2,56*_ = 0.11, *P* = 0.99; time × group: *F*_*2,56*_ = 0.48, *P* = 0.62). Conversely, they decreased significantly for the CS− in all groups (time: *F*_*1,56*_ = 132.27, *P* < 0.001, *η*^*2*^_*p*_ = 0.70; group: *F*_*2,56*_ = 0.97, *P* = 0.39; time × group: *F*_*2,56*_ = 0.75, *P* = 0.48). Thus, participants learned that the CS+ but not the CS− would be followed by the US1. Interestingly, the expectancies to receive the US2 decreased significantly for the CS− (time: *F*_*1,56*_ = 73.60, *P* < 0.001, *η*^*2*^_*p*_ = 0.57; group: *F*_*2,56*_ = 0.75, *P* = 0.48; time × group: *F*_*2,56*_ = 0.15, *P* = 0.87), but remained the same from before to after conditioning for the CS+ in all three groups (time: *F*_*1,56*_ = 0.08, *P* = 0.77; group: *F*_*2,56*_ = 0.10, *P* = 0.91; time × group: *F*_*2,56*_ = 0.17, *P* = 0.85, see Fig. 3). These results suggest that participants in all three groups remained uncertain about a potential switch of CS+ outcomes despite not having experienced any CS+ US2 pairing during conditioning.

#### Day 2-memory reactivation

Groups did not differ in their expectancies to receive the US1 or US2 for the CS+ or the CS− before the start of the reactivation session (for complete results see Supplementary Table 4). Expectancy ratings from before to after memory reactivation on day 2 did show a significant change that differed between groups, stimuli and USs, though (group × stimulus × US × time: *F*_*1,57*_ = 13.83, *P* < 0.001, *η*^*2*^_*p*_ = 0.33; for complete results see Supplementary Table 4). There was no significant change of US1 expectancies (stimulus: *F*_*1,19*_ = 325.55, *P* < 0.001, *η*^*2*^_*p*_ = 0.94, time: *F*_*1,19*_ = 0.07, *P* = 0.79, stimulus × time: *F*_*1,19*_ = 2.32, *P* = 0.14) or US2 expectancies (stimulus: *F*_*1,19*_ = 16.69, *P* < 0.001, *η*^*2*^_*p*_ = 0.47, time: *F*_*1,19*_ = 0.30, *P* = 0.59, stimulus × time: *F*_*1,19*_ = 1.25, *P* = 0.28) for CS+ and CS− in the control group, confirming that the control group did not update their US1 or US2 expectancies due to a lack of PE during reactivation.

In the *value-PE group*, expectancies to receive the US1 changed from before to after reactivation (stimulus: *F*_*1,19*_ = 27.09, *P* < 0.001, *η*^*2*^_*p*_ = 0.59, time: *F*_*1,19*_ = 0.02, *P* = 0.88, stimulus × time: *F*_*1,19*_ = 11.75, *P* < 0.001, *η*^*2*^_*p*_ = 0.38) with expectancies for the CS+ decreasing significantly (pre- vs. post: *t*_*19*_ = − 3.64, *P* = 0.002, *d* = 0.81) as intended by the omission of the US1. Expectancies for the CS− on the other hand increased significantly (*t*_*19*_ = 2.48, *P* < 0.02, *d* = 0.55), showing that the omission of the US1 in the value-PE group may have increased uncertainty about the safety of the CS−. Expectancies to receive the US2 did not differ significantly between CS+ and CS− and did not change significantly from before to after reactivation in the value-PE group (stimulus: *F*_*1,19*_ = 2.70, *P* = 0.12, time: *F*_*1,19*_ = 0.18, *P* = 0.68, stimulus × time: *F*_*1,19*_ = 1.93, *P* = 0.18).

Lastly, the *identity-PE group* changed both their US1 expectancies (stimulus: *F*_*1,19*_ = 162.02, *P* < 0.001, *η*^*2*^_*p*_ = 0.90, time: *F*_*1,19*_ = 1.90, *P* = 0.18, stimulus × time interaction: *F*_*1,19*_ = 8.64, *P* = 0.008, *η*^*2*^_*p*_ = 0.31) and US2 expectancies differently for CS+ and CS− (stimulus: *F*_*1,19*_ = 112.35, *P* < 0.001, *η*^*2*^_*p*_ = 0.86, time: *F*_*1,19*_ = 10.76, *P* = 0.004, *η*^*2*^_*p*_ = 0.36, stimulus × time interaction: *F*_*1,19*_ = 33.33, *P* < 0.001, *η*^*2*^_*p*_ = 0.64). After the unexpected delivery of the US2 for the CS+, expectancies to receive the US2 for the CS+ increased (*t*_*19*_ = 5.17, *P* < 0.001, *d* = 1.16) whereas expectancies to receive the US2 decreased significantly for the CS− (*t*_*19*_ = − 3.25, *P* = 0.004, d = 0.73). Expectancies to receive the US1 for the CS+ decreased significantly (pre- vs. post: *t*_*19*_ = − 2.53, *P* = 0.02, *d* = 0.57) and did not change for the CS− (*t*_*19*_ = 1.67, *P* = 0.11). These results confirm that the unexpected delivery of the US2 in the identity-PE group led to higher expectations to receive the US2, but at the same time updated expectations to receive the US2 for the CS− and expectations to receive the US1 for the CS+.

#### Day 3-memory retention and reinstatement test

The group differences were sustained on day 3 and expectancy ratings assessed before the start of the experiment showed a significant stimulus × US × group interaction (*F*_*2,57*_ = 9.48, *P* < 0.001, *η*^*2*^_*p*_ = 0.25) with significant group differences in expectancies for US1 (*F*_*2,57*_ = 6.44, *p* = 0.003, *η*^*2*^_*p*_ = 0.18) and US2 (*F*_*2,57*_ = 12.96, *P* < 0.001, *η*^*2*^_*p*_ = 0.31) for the CS+, but not the CS− (US1: *F*_*2,57*_ = 2.03, *P* = 0.14; US2: *F*_*2,57*_ = 1.12, *P* = 0.33). Specifically, the value-PE group showed significantly lower expectancies to receive the US1 to the CS+ than the control group (*t*_*38*_ = 3.21, *P* = 0.003, *d* = 1.05), whereas US1 expectancies between value-PE and identity-PE group (*t*_*38*_ = 1.98, *P* = 0.06) and between the control and the identity-PE group did not differ significantly (*t*_*38*_ = 1.87, *P* = 0.07). The identity-PE group did, however, show significantly higher expectancies to receive the US2 to the CS+ than both the control (*t*_*38*_ = 4.88, *P* < 0.001, d = 1.54), and the value-PE group (*t*_*38*_ = 4.66, *P* < 0.001, *d* = 1.47). Here, value-PE and control group did not differ (*t*_*38*_ = 0.41, *P* = 0.69). There were no significant group differences in respect to US expectancies after the experimental session anymore. Summarizing, US expectancy ratings generally confirmed the success of the experimental manipulations and the groups updated their US1 and US2 expectancies according to the different outcomes they experienced during memory reactivation on day 2.

### Fear potentiated startle responses

#### Day 1-conditioning

**Figure 4:**
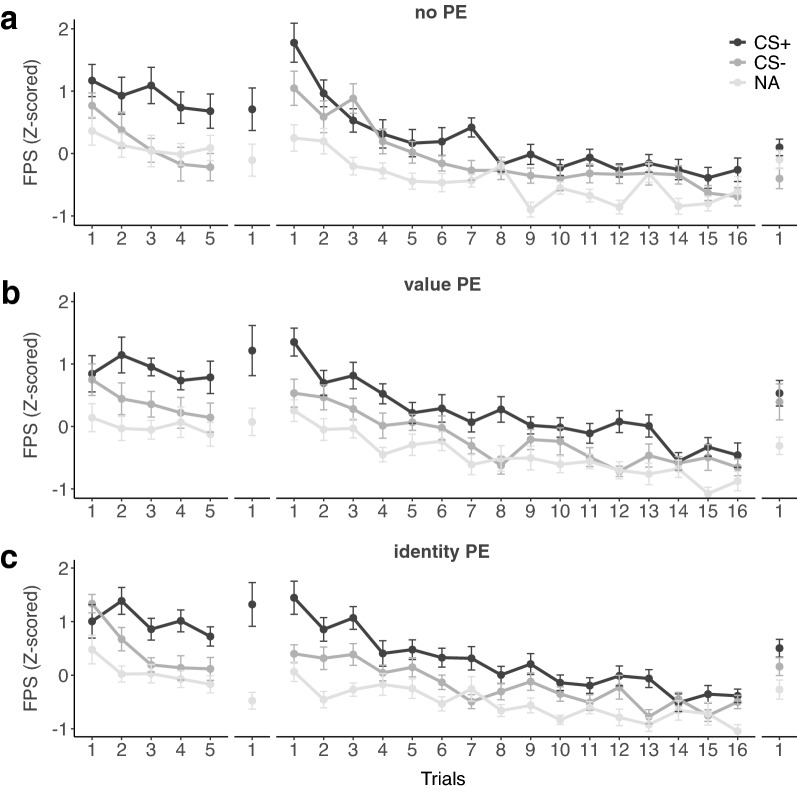
Startle responses during CS+ , CS− and noise alone (NA) trials across conditioning on day 1, fear memory reactivation on day 2, and retention (in extinction) and reinstatement test on day 3 in (**a**) the no-PE control group, (**b**) the value-PE group, and (**c**) the identity-PE group. Error bars depict standard errors of the mean.

Fear potentiated startle responses (see Fig. 4) confirmed that conditioning on day 1 was successful in all groups (stimulus: *F*_*1,55*_ = 12.22, *P* = 0.001, *η*^*2*^_*p*_ = 0.18; trial: *F*_*1,55*_ = 21.71, *P* < 0.001, *η*^*2*^_*p*_ = 0.28; stimulus × trial: *F*_*1,55*_ = 5.78, *P* = *0.0*2, *η*^*2*^_*p*_ = 0.10; group: *F*_*2,55*_ = 0.47, *P* = 0.63; stimulus × group: *F*_*2,55*_ = 1.78; *P* = 0.18, trial × group: *F*_*2,55*_ = 1.13, *P* = 0.33; stimulus × trial × group: *F*_*2,55*_ = 0.25, *P* = 0.78). Specifically, startle responses did not differ between CS+ and CS− on the first trial of conditioning (*t*_*57*_ = 0.33, *P* = 0.74), but were significantly larger for the CS+ than the CS− on the last trial of conditioning (*t*_*57*_ = 3.89, *P* < 0.001, d = 0.71). The exact electrode location (wrist vs. ankle) did not affect the success of conditioning (electrode location: *F*_*1,55*_ = 3.21, *P* = 0.08; stimulus: *F*_*1,55*_ = 15.06, *P* < 0.001, *η*^*2*^_*p*_ = 0.21; electrode location × stimulus: *F*_*1,55*_ = 2.30, *P* = *0.1*4).

#### Day 2-memory reactivation

During memory reactivation on day 2, we observed significantly greater FPS to CS+ than the NA (stimulus: *F*_*1,55*_ = 22.97, *P* < 0.001, *η*^*2*^_*p*_ = 0.29) in all three groups (group: *F*_*2,55*_ = 0.66, *P* = 0.52; stimulus × group: *F*_*2,55*_ = 1.20, *P* = 0.31) confirming that fear memories were successfully retrieved.

#### Day 3-memory retention and reinstatement test

**Figure 5:**
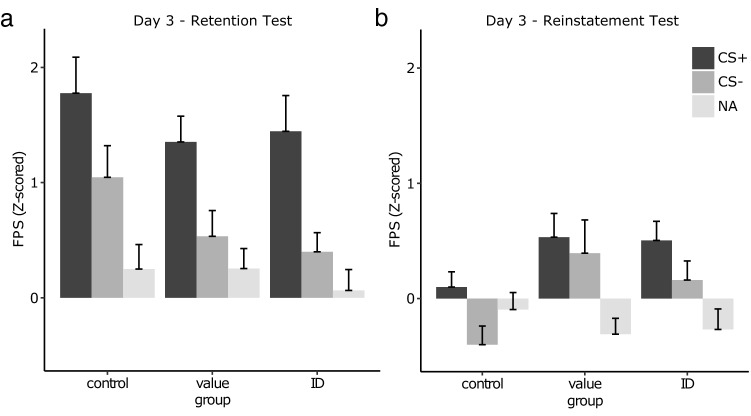
Comparisons of startle responses (Z-scored) during CS+, CS− and noise alone (NA) trials on (**a**) the first trial of the retention test and (**b**) the reinstatement test on day 3 did not reveal any significant group differences. Error bars depict standard errors of the mean.

On the first retention trial on day 3, FPS were significantly greater to the CS+ than to the CS− (stimulus: *F*_*1,57*_ = 19.69, *P* < 0.001, *η*^*2*^_*p*_ = 0.26). In contrast to our hypothesis, this effect did, however, not differ between groups (group: *F*_*2,57*_ = 2.03, *P* = 0.07; stimulus × group: *F*_*2,57*_ = 0.23, *P* = 0.79; see Fig. 5). In contrast to previous findings^8,9,38^, propranolol administration after a value-PE did not reduce the expression of fear compared to a no-PE control group (stimulus × group: *F*_*1,38*_ = 0.04, *P* = 0.85). Fear expression did also not differ between the identity-PE and the value-PE group (stimulus × group: *F*_*1,38*_ = 0.22, *P* = 0.64) and the identity-PE and the no-PE group (stimulus × group: *F*_*1,38*_ = 0.45, *P* = 0.51). Differential startle responses decreased significantly from the first to the last trial of extinction (stimulus: *F*_*1,57*_ = 24.92, *P* < 0.001, *η*^*2*^_*p*_ = 0.30; trial: *F*_*1,57*_ = 149.95, *P* < 0.001, *η*^*2*^_*p*_ = 0.72; stimulus × trial: *F*_*1,57*_ = 6.32, *P* = *0.0*2, *η*^*2*^_*p*_ = 0.10) and there were no group differences in respect to extinction (group: *F*_*2,57*_ = 1.82, *P* = 0.17; stimulus × group: *F*_*2,57*_ = 0.05, *P* = 0.95; trial × group: *F*_*2,57*_ = 1.46, *P* = 0.24; stimulus × trial × group: *F*_*2,57*_ = 0.55, *P* = 0.58). The unannounced delivery of the US1 induced a significant increase of FPS to both CS+ and CS− (i.e., non-differential reinstatement; stimulus: *F*_*1,57*_ = 6.95, *P* = 0.01, *η*^*2*^_*p*_ = 0.11; trial: *F*_*1,57*_ = 42.91, *P* < 0.001, *η*^*2*^_*p*_ = 0.43; stimulus × trial: *F*_*1,57*_ = 0.20, *P* = *0.6*6) that differed significantly between groups (group: *F*_*2,57*_ = 3.31, *P* = 0.04, *η*^*2*^_*p*_ = 0.10; trial × group: *F*_*2,57*_ = 3.57, *P* = 0.04, *η*^*2*^_*p*_ = 0.11; stimulus × group: *F*_*2,57*_ = 0.69, *P* = 0.50; stimulus × trial × group: *F*_*2,57*_ = 0.21, *P* = 0.81). Whereas there was no group difference at the end of extinction (stimulus: *F*_*1,57*_ = 3.57, *P* = 0.06; group: *F*_*2,57*_ = 0.31, *P* = 0.73; stimulus × group: *F*_*2,57*_ = 0.53, *P* = 0.59), groups differed after reinstatement (stimulus: *F*_*1,57*_ = 4.63, *P* = 0.04; group: *F*_*2,57*_ = 5.22, *P* = 0.008, *η*^*2*^_*p*_ = 0.15; stimulus × group: *F*_*2,57*_ = 0.47, *P* = 0.63). This effect was mainly driven by smaller CS− responses in the control compared to the two other groups (control vs. value-PE: *t*_*38*_ = − 2.40, *P* = 0.02, d = 0.76; control vs. identity-PE: *t*_*38*_ = − 2.42, *P* = 0.02, d = 0.76). Responses to the CS+ did not differ between control and the other groups (all *t’s* < 1.91, *P’s* > 0.06), and value-PE and identity-PE group did not differ significantly from each other irrespective of CS+ or CS− responses (all *t’s* < 0.70, *P’s* > 0.50). Summarizing, in contrast to our hypothesis, neither prediction error manipulation reduced conditioned responses at retention or reinstatement test significantly.

### Post-experiment US ratings

When asked whether they still remembered the two USs as equally uncomfortable after the end of the experiment on day 3, only 50% of participants, i.e., 30 out of 60, indeed did (see Supplementary Table 5). In order to exclude that the results were affected by differences in US-value, we computed the main analyses in the sub-sample of participants that still perceived US1 and US2 as equally aversive. Excluding participants who did not remember the USs as equally aversive did not affect the results at retention or reinstatement test (see Supplementary Table 6). Among the 50% of participants (N = 30) who did not remember the USs as equally aversive, N = 16 participants reported remembering the US1 and N = 14 participants reported remembering the US2 as more aversive (*χ*^2^ = 0.13, *P* = 0.72), suggesting that whether or not a US was used for conditioning did not significantly affect participant’s later judgement of aversiveness. Among those participants, there was, however, a bias towards remembering the electric stimulus to the arm as more aversive than the stimulus to the leg (*χ*^2^ = 8.53, *P* = 0.004, *d* = 1.26), suggesting that the exact electrode location affected (the memory of) the subjective aversiveness of the two electric stimuli in a sub-set of participants.

## Discussion

The present study aimed to test the effect of different types of prediction errors in combination with a pharmacological disruption of fear memory reconsolidation on fear memory expression. Specifically, we investigated whether a model-based ‘identity’ PE is sufficient or a ‘value’ PE necessary to render fear memories susceptible to reconsolidation disruption. In contrast to our expectations and previous findings (see for example ref.^6,9^), we could not replicate the fear-reducing effect of a pharmacological reconsolidation disruption after a value PE. Instead, the value-PE group showed sustained differential startle responding comparable to a control group in which no PE was elicited during memory reactivation. Similarly, the identity-PE group continued to show differential startle responses at the beginning of the retention and after reinstatement test. The lack of reconsolidation disruption in the value-PE group does, however, make an interpretation of the results in the identity-PE group difficult. Our results do not confirm previous results of successful disruption of fear memory reconsolidation with propranolol^6–12,25,30,41^ but are rather in line with a small number of studies that could not replicate the effect^42–44^.

A potential explanation for the lack of reconsolidation disruption in the value-PE group may be their lack of certainty about the CS–US contingency. Specifically, we had calibrated two USs, US1 and US2, to arm and leg in all three groups. Expectancy ratings indicated that the omission of the US1 in the value-PE group did indeed result in a decrease of US1 expectancy for the CS+ , as in previous studies. At the same time, however, expectancies of a pairing of the CS+ (and CS−) with the US2 were still substantially greater than zero and only dropped after the end of the experimental session on day 3. Furthermore, the omission of the US1 in the value-PE group may have increased uncertainty about the safety of the CS−, as indicated by an increase in CS− expectancies from before to after reactivation in that group. Thus, even though the value-PE group had experienced a clear value PE as in previous studies (see for example ref.^6,9^), uncertainty surrounding a potential delivery of the US2 and uncertainty about the CS–US contingency may have prevented an actual destabilization of the fear memory.

Of note, there are a number of other paradigm differences between the present and previous studies. Next to sample differences (present study: international students, past study: primarily Dutch students) and a sex difference in experimenters (present: male experimenter, past: female experimenter), the stimuli employed as CSs and the employed expectancy ratings differed between studies. Compared to previous studies that used a fear-relevant stimulus (e.g., picture of spider or gun) as CS and did observe successful reconsolidation disruption^6–11,25,30,31^, we here employed fear-irrelevant CSs (i.e., abstract fractals). Previous work suggests that the usage of fear-irrelevant conditioned stimuli may result in weaker fear memories^45^, while weak and strong fear memories may require different forms of reactivation to trigger memory reconsolidation^46^. It is thus conceivable that the single omission of the US (after conditioning with full reinforcement) may have been optimal to trigger reactivation in a strong fear memory in past studies, but already induced extinction or a transitional limbo-state^47^ in the ‘weaker’ fear memory in the value-PE group of the present study. Alternatively, the different form of expectancy rating employed here may have affected the results. In the present study, we assessed expectancy ratings pre- and post-session instead of online during each trial. Online expectancy ratings may increase attention to the violation of the expected US delivery and thereby amplify the value PE and facilitate fear memory destabilization. Thus, before not having replicated the value-PE effect in this new paradigm, we cannot attribute the lack of reconsolidation disruption in the present study to the uncertainty around CS–US contingencies, but can only speculate that it may have hindered fear memory destabilization.

Generally, an open question is whether the lack of fear reduction after a value PE and propranolol administration in the present study was due to failed memory destabilization or a lack of an effect of propranolol on memory reconsolidation. We did observe a decrease in blood pressure from before to after propranolol intake that was comparable to that reported by previous studies which strongly suggests that propranolol exerted an effect. However, the lack of a placebo control group-a clear limitation of the present study design-does not allow us to finally exclude that a lack of an effect of propranolol can account for the lack of fear reduction in the present study. Independent of the failure to replicate the desired effect in the value-PE group, the present paradigm seemed generally suitable to induce a model-based identity PE. Namely, the success of fear conditioning did not differ significantly between participants who received a US to the wrist compared to the ankle and pre- and post-conditioning ratings of US aversiveness were not affected by whether a US to wrist or arm was delivered during conditioning. Furthermore, US expectancy ratings in the identity-PE group were in line with the experimental manipulation. However, in post-experiment interviews, only 50% of participants indicated that they still remembered both USs as equally uncomfortable and electric stimuli to the wrist were remembered as more aversive as stimuli to the ankle, despite having been calibrated to a level perceived as equally aversive. Thus, future research aiming to equalize electric stimuli in terms of aversive value should consider using the electric stimuli to the same but contralateral body location.

Understanding the exact types of PEs that destabilize fear memories is a fundamental challenge of future research on reconsolidation-based interventions. Previous research has already determined that, specifically, positive and negative value-PEs and temporal PEs can make associative fear memories in humans and animals susceptible to manipulations^14^. Whether or not model-based Pes-elicited by unexpected US-properties beyond value or temporal mismatches-also destabilize emotional memories remains elusive. If model-based PEs turn out to be sufficient for memory destabilization, this could have important implications for the creation of clinical settings that allow for emotional memory change (e.g., not a necessity to create outcomes better than expected). It is, however, more likely that—as long as not accompanied by PEs in a model-free value learning system—model-based PEs are merely sufficient to induce new learning and changes in some aspects of the memory (e.g., CS–US contingency knowledge), but would leave the affective component of the emotional memory intact.

Summarizing, the present study aimed to compare the effect of a pharmacological reconsolidation disruption after no PE, a value-PE and a purely model-based identity PE. No evidence for successful reconsolidation disruption after a model-based identity PE compared to no PE was found. However, in contrast to previous studies, reconsolidation disruption in combination with a value PE did also not result in a reduction of fear. This failure to replicate a pharmacological disruption of fear memory reconsolidation can potentially be explained by an increased uncertainty of participants about CS–US contingencies after fear memory reactivation in the present study design but its exact cause remains unknown.

## Supplementary Information

Supplementary Information.

## Data Availability

The data that support the findings of this study are openly available at https://doi.org/10.17605/OSF.IO/8XM3H.
