# Supplementary material for: Better, worse, or different than expected: on the role of value and identity prediction errors in fear memory reactivation
Source: Sci Rep. 2022 Apr 7;12:5862. doi: 10.1038/s41598-022-09720-w (PMC8988102; doi:10.1038/s41598-022-09720-w)
Supplement: Supplementary file 1 — Supplementary Information. [file 41598_2022_9720_MOESM1_ESM.pdf]

SUPPLEMENTARY MATERIAL

Gerlicher AMV, Verweij SA, Kindt M. Better, worse, or different than expected - On the role of value and identity prediction errors in fear memory reactivation.

Supplementary Table 1. Table illustrating which type of prediction error (i.e., model-free, model-based) was elicited by experimental manipulations during memory reactivation in the cited reconsolidation studies. The color code indicates whether these prediction-error conditions allowed for the destabilization and successful reconsolidation disruption of fear memories (green) or not (red). This perspective seems to suggest that a co-occurrence of both model-free and model-based prediction error may be a prerequisite to destabilize fear memories. However, before drawing any firm conclusions, the missing condition (model-based but no model-free prediction error) requires empirical testing.

		**Model-free prediction error?**	
		Yes	No
**Model-based prediction error?**	Yes	Unexpected omission of US[1]<br><br>US at unexpected time[2]	?
	No	Unexpected omission of US in absence of US electrode[3]	No PE[1]

Results of Day 3 FPS Analysis after Exclusion of Data of N=2 Participants

Excluding data of two participants who did not contribute day 1 and day 2 FPS data due to a technical failure with the recording software, does not affect the results on day 3. On the first retention trial on day 3, FPS were still significantly greater to the CS+ than to the CS- (stimulus: $F_{1,55}$=20.21, p<.001, η^2_p=.27) in all groups (group: $F_{2,55}$=1.20, p=.31; stimulus x group: $F_{2,55}$=.81, p=.45). Differential startle responses decreased significantly from the first to the last trial of extinction (stimulus: $F_{1,55}$=26.54, p<.001, η^2_p=.33; trial: $F_{1,55}$=154.60, p<.001, η^2_p=.74; stimulus x trial: $F_{1,55}$=5.78, p=.02, η^2_p=.10) and there were no group differences in respect to extinction (group: $F_{2,55}$=1.19, p=.31; stimulus x group: $F_{2,55}$=.25, p=.78; trial x group: $F_{2,55}$=73, p=.48; stimulus x trial x group: $F_{2,55}$=.97, p=.39). The unannounced delivery of the US1 induced a significant increase of FPS to both CS+ and CS- (i.e., non-differential reinstatement; stimulus: $F_{1,55}$=7.18, p=.01, η^2_p=.12; trial: $F_{1,55}$=42.91, p<.001, η^2_p=.44; stimulus x trial: $F_{1,55}$=.20, p=.66) that differed significantly between groups (group: $F_{2,55}$=2.93, p=.06, η^2_p=.10; trial x group: $F_{2,55}$=3.40, p=.04, η^2_p=.11; stimulus x group: $F_{2,55}$=.54, p=.59; stimulus x trial x group: $F_{2,55}$=.22, p=.81). There was no group difference at the end of extinction (stimulus: $F_{1,55}$=3.66, p=.06; group: $F_{2,55}$=.18, p=.84; stimulus x group: $F_{2,55}$=.36, p=.70), but groups differed after reinstatement (stimulus: $F_{1,55}$=4.73, p=.03, η^2_p=.08; group: $F_{2,55}$=4.82, p=.01, η^2_p=.15; stimulus x group: $F_{2,55}$=.44, p=.65). This effect was still driven by smaller CS- responses in the control compared to the two other groups (control vs. value-PE: t_{37}=-2.30, p=.03, d=.73; control vs. identity-PE: t_{36}=-2.16, p=.04, d=.70). Responses to the CS+ did not differ between control and the other groups (all *t's*<2.03, *p's*>.05), and the value-PE and identity-PE group did not differ significantly from each other irrespective of CS+ or CS- responses (all *t's*<.75, *p's*>.46).

Supplementary Table 2. Mean (standard deviation) heart rate (HR) and blood pressure (BP, systolic/diastolic) assessed before the start of the experiment on day 1, and before and after the experiment on day 2 in the three experimental groups. F- and p-values refer to results of group comparisons by rmANOVA.

	no PE	MFMB PE	MB PE	*F*-value	*p*-value
Day 1 HR pre	82.1 (12.4)	78.9 (12.3)	82.7 (14.4)	.49	.61
Day 1 BP pre	119.0/79.2 (18.0/10.3)	118.9/74.8 (13.4/9.9)	116.3/77.0 (10.4/12.2)	.22/.80	.80/.45
Day 2 HR pre	84.0 (14.4)	82.7 (18.5)	83.7 (12.9)	.04	.97
Day 2 BP pre	118.2/76.8 (14.0/7.9)	119.2/71.3 (12.4/17.7)	119.8/73.2 (10.8/8.6)	.08/1.0	.92/.35
Day 2 HR post	62.6 (8.9)	62.9 (10.5)	63.9 (10.7)	.09	.92
Day 2 BP post	103.3/70.8 (10.2/7.1)	109.8/67.3 (23.4/9.7)	103.2/70.8 (24.6/9.3)	.68/1.1	.51/.35

Supplementary Table 3. Average decrease of systolic/diastolic blood pressure (BP) and heart rate in propranolol HCl treated participants (values were averaged over propranolol conditions in each study).

Author/Year	Δ systolic BP	Δ diastolic
Kindt et al. (2009)[4]	14.45	6.8
Kindt & Soeter (2018)[5]	N/A	N/A
Sevenster et al. (2012)[3]	12.99	4.69
Sevenster et al. (2013)[1]	13.26	4.37
Sevenster et al. (2014)[6]	12.09	2.67
Soeter & Kindt (2010)[7]	16.28	4.83
Soeter & Kindt (2011)[8]	19.64	5.6
Soeter & Kindt (2012)[9]	22.5	6.1
Soeter & Kindt (2015)[10]	15.1	5.8
Soeter & Kindt (2015) [11]	N/A	N/A

Supplementary Table 4. Complete results for repeated measures ANOVAs on pre-/post-session US expectancy ratings on day 1, day 2 and day 3 with the indicated within- and between-subject factors.

	group	stimulus	US	time	group x stimulus	group x US	group x time	stimulus x US	stimulus x time	US x time	group x stimulus x US	group x stimulus x time	stimulus x US x time	group x US x time	group x stimulus x US x time
Day 1	$F_{2,56}$=.30 p=.75	$F_{1,56}$=168.82 p<.001	$F_{1,56}$=51.63 p<.001	$F_{1,56}$=8.95 p=.004	$F_{2,56}$=.91 p=.41	$F_{2,56}$=.16 p=.85	$F_{2,56}$=.14 p=.87	$F_{1,56}$=65.46 p<.001	$F_{1,56}$=166.82 p<.001	$F_{1,56}$=35.40 p<.001	$F_{2,56}$=.42 p=.66	$F_{2,56}$=.61 p=.55	$F_{1,56}$=69.91 p<.001	$F_{2,56}$=.00 p=.99	$F_{2,56}$=.42 p=.66
Day 2 pre	$F_{2,57}$=2.48 p=.09	$F_{1,57}$=197.26 p<.001	$F_{1,57}$=27.13 p<.001	-	$F_{2,57}$=1.12 p=.33	$F_{2,57}$=1.42 p=.25	-	$F_{1,57}$=121.01 p<.001	-	-	$F_{2,57}$=.16 p=.86	-	-	-	-
Day 2	$F_{2,57}$=3.00 p=.06	$F_{1,57}$=185.88 p<.001	$F_{1,57}$=31.18 p<.001	$F_{1,57}$=1.61 p=.21	$F_{2,57}$=6.28 p=.003	$F_{2,57}$=5.05 p=.01	$F_{2,57}$=.36 p=70	$F_{1,57}$=107.36 p<.001	$F_{1,57}$=1.25 p=.29	$F_{1,57}$=4.64 p=.04	$F_{2,57}$=7.69 p=.001	$F_{2,57}$=10.18 p<.001	$F_{1,57}$=13.85 p<.001	$F_{2,57}$=2.30 p=.11	$F_{2,57}$=13.85 p<.001

Supplementary Table 5. Results of post-experiment questions about the aversiveness of the two electric stimuli.

	no PE	MFMB PE	MB PE
Do you still remember the two stimuli as equally uncomfortable? - Yes/**No**	10/**10**	8/**12**	12/**8**
If not, which of the two stimuli do you remember as more uncomfortable?	N=6 US1<br>N=4 US2<br><br>N=10 arm	N=6 US<br>N=6 US2<br><br>N=9 arm<br>N=3 leg	N=4 US1<br>N=4 US2<br><br>N=4 arm<br>N=4 leg

Supplementary Table 6. Results of repeated measures ANOVA with the indicated within- and between-subject factors on fear potentiated startle responses of a sub-sample of N=30 participants that indeed remembered the US1 and US2 still as equally aversive after the end of the experiment on day 3 (see Supplementary Table 4).

	group	stimulus	time	group x stimulus	group x time	stimulus x time	group x stimulus x time
Day 1 - conditioning	$F_{2,26}$=1.71 p=.20	$F_{1,26}$=7.78, p=.01	$F_{1,26}$=8.03, p=.009	$F_{2,26}$=.58 p=.57	$F_{2,26}$=1.55 p=.23	$F_{1,26}$=8.15, p=.008	$F_{2,26}$=.12 p=.88
Day 2 - retrieval	$F_{2,26}$=.50 p=.61	$F_{1,26}$=39.87, p<.001	-	$F_{2,26}$=.11 p=.90	-	-	-
Day 3 – retention	$F_{2,27}$=2.89 p=.07	$F_{1,27}$=9.27, p=.005	-	$F_{2,27}$=.26 p=.77	-	-	-
Day 3 – extinction	$F_{2,27}$=4.63 p=.02	$F_{1,27}$=12.59, p=.001	$F_{1,27}$=81.03, p<.001	$F_{2,27}$=.09 p=.91	$F_{2,27}$=1.13 p=.34	$F_{1,27}$=3.47, p=.07	$F_{2,27}$=.43 p=.65
Day 3 – reinstatement	$F_{2,27}$=2.33 p=.12	$F_{1,27}$=12.63, p=.001	-	$F_{2,27}$=.66 p=.53	-	-	-
